# A158 COMPARING CORTIMENT® AND PREDNISONE IN ULCERTATIVE COLITIS: A POPULATION-BASED STUDY OF OUTCOMES

**DOI:** 10.1093/jcag/gwab049.157

**Published:** 2022-02-21

**Authors:** S Coward, K Martins, S Klarenbach, K Kroeker, C Ma, R Panaccione, L Richer, C Seow, L E Targownik, G G Kaplan

**Affiliations:** 1 Internal Medicine, University of Manitoba, Winnipeg, MB, Canada; 2 University of Alberta, Edmonton, AB, Canada; 3 University of Calgary, Calgary, AB, Canada

## Abstract

**Background:**

In August 2016 Cortiment® was approved for use in ulcerative colitis (UC) patients in Canada, but not approved for reimbursement; the Canadian Agency for Drugs and Technology in Health cited no comparable benefit for its use over other approved UC medications. Real-world data comparing Cortiment® to other UC medications is limited, especially during the COVID-19 pandemic where the use of steroids is counter-indicated for COVID-19-related outcomes.

**Aims:**

To examine the comparative risk of hospitalization, surgery, and infection after initiation of Cortiment® or oral corticosteroids among UC patients using real-world data

**Methods:**

Using population-based data from Alberta Canada, two cohorts were compared: 1. Patients dispensed Cortiment® and an ICD diagnostic code for UC [9: 556.X; 10: K51.X] (August 1, 2016 to October 31, 2019); and, 2. Validated (algorithm) UC patients dispensed a >30 day supply or >500mg in 24 hours of prednisone/prednisolone (April 1, 2016 to October 31, 2019). All hospitalizations, IBD-surgery, or infections (i.e., pneumonia, c.diff, sepsis, tuberculosis) that occurred 6 or 12 months from initial medication dispensing were identified. Cox-proportional hazard models, with Hazard Ratios (HR), assessed comparative outcomes. Kaplan-Meier survival curves were created, and Poisson regression (or negative binomial) used to assess the Average Monthly Percentage Change (AMPC) with associated 95% confidence intervals (CI).

**Results:**

We identified 917 Cortiment® and 2,404 Prednisone patients. Over the study period, prednisone dispensing significantly decreased (AMPC:-2.53% [CI:-2.85,-2.21]) while Cortiment® remained stable. Dispensing of Cortiment® significantly decreased the hazard of hospitalization (all types, except surgery) at 12 months as compared to prednisone, and significantly decreased the hazard of an infection at both 6 and 12 months (Table 1, Fig 1).

**Conclusions:**

The use of Cortiment® in a real-world setting is associated with fewer deleterious outcomes, and its use during a pandemic should be preferred, especially when it’s counterpart can exacerbate negative COVID-19-related outcomes.

Table 1

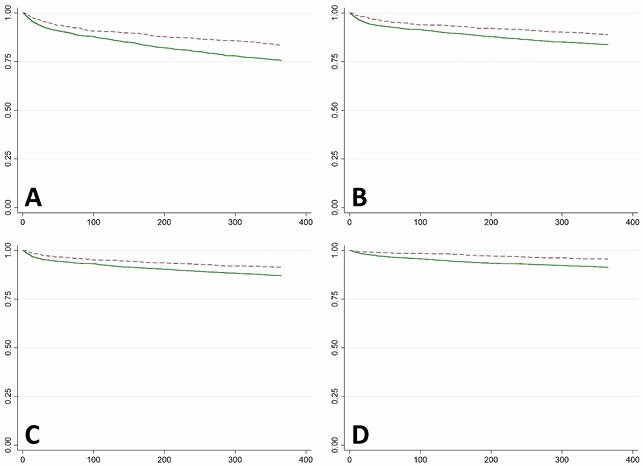

Kaplan-Meier Survival Curves of 1-year Outcomes: A) All Hospitalizations; B) IBD-Related Hospitalizations; C) IBD-Specific Hospitalizations; and, D) Any Infection.

**Dashed Line:**

Cortiment Cohort

**Solid Line:**

Prednisone/Prednisolone Cohort

**Funding Agencies:**

Ferring Pharmaceuticals

**Table:** 

		Cortiment (%)	Prednisone (%)	
	N	917	2,404	
Age	Median (Q1; Q3)	45.9 (33.3, 58.5)	43.9 (31.8, 59.1)	
Sex	Male	430 (46.9)	1,356 (56.4)	
Female	487 (53.1)	1,048 (43.6)	
		Cortiment; n(%)	Prednisone; n (%)	HR (95%CI)
All Hosp	6-months	107 (11.67)	409 (17.0)	0.83 (0.63, 1.11)
1-year	152 (16.58)	592 (24.6)	0.64 (0.54, 0.77)
IBD-Related	6-months	72 (7.85)	279 (11.6)	0.67 (0.52, 0.87)
1-year	102 (11.12)	394 (16.4)	0.66 (0.53, 0.82)
IBD-Specific	6-months	58 (6.32)	223 (9.28)	0.91 (0.63, 1.32)
1-year	79 (8.62)	312 (12.98)	0.66 (0.51, 0.84)
IBD-Surgery	6-months	13 (1.42)	57 (2.37)	0.61 (0.33, 1.11)
1-year	24 (2.62)	101 (4.20)	1.05 (0.61, 1.82)
Infection	6-months	25 (2.73)	152 (6.32)	0.43 (0.28, 0.65)
1-year	41 (4.47)	213 (8.86)	0.49 (0.35, 0.69)

